# Using an adaptive trial design for an infectious disease in primacy care - challenges in the design and set-up stages

**DOI:** 10.1186/1745-6215-16-S2-P211

**Published:** 2015-11-16

**Authors:** Johanna Cook, Theo Verheij, Alike van der Velden, Herman Goossens, Menno de Jong, Philippe Beutels, Paul Little, Chris Butler

**Affiliations:** 1 University of Oxford, Oxford, UK; 2 University of Southampton, Southampton, UK; 3 University Medical Centre Utrecht, Utrecht, The Netherlands; 4 University of Amsterdam, Amsterdam, The Netherlands; 5 University of Antwerp, Antwerp, Belgium

## 

Conducting trials of infectious disease is challenging given the short duration of the disease and the potential for change in the condition. The ALIC^4^E Trial (Antivirals for influenza-Like Illness? an rCt of Clinical and Cost-effectiveness in primary CarE) will investigate the clinical and cost-effectiveness of adding antivirals to best usual primary care for the treatment of influenza-like illness (ILI) using an open, adaptive design trial.

ALIC^4^E is a large pan-Europe trial recruiting 4500 participants in 20 European countries. This will be the first large adaptive design trial of infectious diseases in primary care. It will initially look at usual care versus usual care plus oseltamivir. The adaptive design will allow us to incorporate new drugs as they come on the market and be more responsive to new pandemics as we can incorporate new government initiatives. In addition it will allow us to analyse reactions to treatment within subgroups according to age, duration and severity of illness and the presence of co-morbidities as the trial progresses. The open trial design ensures that the study findings will approximate most closely to real world conditions of clinical care.

ALIC^4^E will be the first large-scale, international, non-industry sponsored trial of cost-effectiveness in primary care of oseltamivir. It will utilise a network of primary care centres across Europe to recruit but will have the challenge of recruitment being limited to confirmed flu season to increase the likelihood of persons with real flu being recruited.

